# HDL protects against myocardial ischemia reperfusion injury via miR-34b and miR-337 expression which requires STAT3

**DOI:** 10.1371/journal.pone.0218432

**Published:** 2019-06-20

**Authors:** Sarah Pedretti, Marie-Claude Brulhart-Meynet, Fabrizio Montecucco, Sandrine Lecour, Richard W. James, Miguel A. Frias

**Affiliations:** 1 Department of Medical Specialties-Endocrinology, Diabetology, Hypertension and Nutrition, University of Geneva, Geneva, Switzerland; 2 First Clinic of Internal Medicine, Department of Internal Medicine, University of Genoa School of Medicine, Genoa, Italy; 3 IRCCS AOU San Martino—IST, Genoa, Genoa, Italy; 4 Centre of Excellence for Biomedical Research (CEBR), University of Genoa, Genoa, Italy; 5 Hatter Institute for Cardiovascular Research in Africa, Department of Medicine, Faculty of Health Sciences, University of Cape Town, Cape Town, South Africa; 6 Division of Laboratory Medicine, Diagnostics Department, Geneva University Hospitals, Geneva, Switzerland; University of PECS Medical School, HUNGARY

## Abstract

**Purpose:**

High density lipoprotein (HDL) protects against myocardial infarction via mechanisms that remain unclear. STAT3 (signal transducer and activator of transcription 3) plays a key role in HDL-induced cardioprotection. In the heart, microRNAs (miRNAs) are involved in ischemia reperfusion injury. We therefore investigated whether the cardioprotective effect of HDL modulates miRNAs as a downstream target of STAT3 activation.

**Methods:**

STAT3 cardiomyocyte deficient mice (STAT3-KO) and wildtype littermates (STAT3-WT) were submitted to left coronary ligature and reperfused (IR) with or without injection of HDL. Infarct size (IS) was determined and cardiac miRNA expression was evaluated after reperfusion in sham, IR and IR+HDL hearts by microarray analysis.

In vitro, neonatal rat ventricular cardiomyocytes were submitted to hypoxia with or without HDL incubation. Cell viability and miRNA expression were analysed.

**Results:**

*In vivo*, HDL reduced IS from 40.5±4.3% to 24.4±2.1% (p<0.05) in STAT3-WT mice. HDL failed to protect in STAT3-KO mice. In STAT3-WT mice, both miR-34b and miR-337 were increased in IR compared to sham and IR+HDL groups (p<0.05). These miRNAs were not modulated in STAT3-KO mice. *In vitro*, incubation with HDL improved cell viability against hypoxia (p<0.05). The expression of miR-34b and miR-337 was increased by hypoxia and reduced by HDL treatment (p<0.05). In cardiomyocytes transfected with miRNA mimics, HDL failed to improve cell viability against hypoxia.

**Conclusions:**

Our study, performed both *in vivo* and *in vitro*, delineates a novel cardioprotective signalling pathway activated by HDL, involving STAT3-mediated decrease of miR-34b and miR-337 expression.

## Introduction

Historically, high density lipoproteins (HDL) have been identified as a strong negative predictor of cardiovascular events. Their beneficial effect on the cardiovascular system was attributed, in the first instance, to their ability to facilitate cholesterol excretion [1]. Recent evidence attributes more widespread, beneficial actions to the HDL particle, such as antioxidant, anti-inflammatory and anti-apoptotic actions on vascular cells [2]. The direct actions of HDL on the heart have not been extensively investigated although experimental data show that HDL protects from ischemia reperfusion injury (IR) [3,4], hypoxia [5,6], and apoptosis induced by doxorubicin, a potent anti-cancer drug with cardiotoxic side effects [7]. In previous publications, we demonstrated that the protective effect of HDL against oxidative stress and IR is mediated via the activation of the Survivor Activating Factor Enhancement (SAFE) pathway which involves the activation of intracellular signalling factors including Tumour Necrosis Factor alpha (TNFα) and Signal Transducer and Activator of Transcription 3 (STAT3) [6,8]. In the heart, STAT3 has been shown to be beneficial and plays a pro-survival role. Multiple studies including ours have demonstrated the anti-apoptotic role of STAT3 in cardiac conditioning [9].

MicroRNAs (miRNAs) are non-coding sequences of 20–22 nucleotides that can modulate the expression of several genes simultaneously. miRNAs are involved in multiple actions that modulate cell homeostasis. In the heart, miRNAs modulate the response during IR to promote cell survival [10]. Recently, several research groups have reported the involvement of some miRNAs in the pathophysiology of myocardial infarction [11,12]. Additionally, some miRNAs have been shown to be involved in myocardial ischemic conditioning [13]. Despite the research into signalling pathways involving miRNAs during conditioning regulation, the role of STAT3 in miRNAs’ regulation has not been investigated. Most of the studies on STAT3 and miRNA regulation are in cancer cells. While some miRNAs have been demonstrated to directly target STAT3 expression [14], only a few studies showed that STAT3 can modulate target miRNA [15]. Interestingly the HDL particle has been shown to contain miRNAs and transports them to several different cell types [16,17]. This leads to the regulation of the expression of target mRNA [17]. In patients, the miRNA profile of HDL is modified under pathophysiological conditions such as familial hypercholesterolemia and coronary artery disease (myocardial infarction, stable and unstable angina) [16,18]. However, little is known about the effect of HDL treatment on cardiac miRNA expression and potential consequences for protection against IR.

In this study, we evaluated whether HDL protects against IR injury by modulating the miRNA expression as a downstream target of STAT3 activation.

## Materials and methods

### Animals

The investigation conforms to the Guide for the Care and Use of Laboratory Animals published by the US National Institutes of Health (NIH Publication No. 85–23, revised 1996) and has been approved by local ethical committee of the Geneva University Medical School (GE12/3891). Animals were housed and treated in accordance with the Guide for Care and Use of laboratory Animals Eighth Edition, published by the US National Institute of Health Publication. Cardiomyocyte specific STAT3-deficient mice (STAT3-KO) and their wildtype littermates (STAT3-WT) from a C57BL/6 genetic background were obtained from the University of Cape Town (South Africa) as previously described [19]. Male mice aged 12–16 weeks and neonatal Wistar rats (obtained from the University Medical Centre (CMU) animal facility, Medical Faculty, University of Geneva, Geneva, Switzerland.) were used in this study. Mice had *ad libitum* access to water and food (standard diet) and were entrained in a 12 hour light and 12 hour dark cycle. Mouse genotype determined the group attribution; the mice were distributed in the group sequentially.

### HDL isolation

HDL (d = 1.063–1.21g/mL) were isolated by cumulative flotation ultracentrifugation as previously described [20] from a plasma pool provided by healthy volunteers (recruited from the University staff). The volunteers gave written, informed consent in accordance with local ethical committee requirements. After isolation, HDL were dialyzed against phosphate buffered saline containing ethylenediaminetetraacetic acid (EDTA) (0.1mM) and stored at 4°C.

### *In vivo* ischemia reperfusion (IR)

IR was achieved through ligation of the left anterior descending (LAD) coronary artery as described in detail previously [21]. In brief, IRI was analysed after ligation of the left anterior descending (LAD) coronary artery. Mice were anaesthetized with 4% isoflurane, intubated and mechanical ventilation performed (150μl, 120 breaths/min) using a rodent respirator (model 683; Harvard Apparatus). Anaesthesia was maintained with 2% isoflurane delivered in 100% O2 through the ventilator. A thoracotomy was performed and an 8–0 prolene suture was passed under the left anterior descending (LAD) coronary artery. After 45min ischemia, LAD coronary artery occlusion was released and reperfused for 8 or 24h. The mouse was injected with the analgesic buprenorphine (0.1 mg/kg mouse body weight, one subcutaneous injection 30min before the intervention and another 8h after the intervention) and was monitored during the first and 8h after the surgery. Approximately 10% of the mice used in this study died during the experiments; from them approximately 90–95% died during or just after the surgery (option stated and approved by the ethical committee). The mice that survived more than one hour after surgery recovered well and did not showed significant, severe signs of illness following the procedures. As stated, the mice were monitored and if they showed signs of illness (abundant bleeding or dyspnea), they were immediately euthanized under anaesthesia ketamine-xylazine (120mg/kg and 16mg/kg, respectively). After 24h reperfusion the mice were re-anaesthetized with ketamine-xylazine (120mg/kg and 16mg/kg, respectively) and the LAD coronary artery was re-occluded. Evan’s blue dye 2% (Sigma) was injected to delineate the in vivo area at risk (AAR). The hearts were harvested, rinsed with PBS, sliced into 5–6 sections and stained with triphenyltetrazolium chloride (TTC 1%) to allow quantification of infarct size. The different zones were determined using a computerized planimetric technique (MetaMorph v6.0, Universal Imaging Corporation) and IS was expressed in percentage of AAR (I/AAR). We paid particular attention to the quantification of the different areas; each slice was carefully evaluated, as it is known that Evans blue can precipitate out of solution on a subsequent addition of TTC and prone to smearing during slicing, resulting in an ambiguous border definition and could therefore explain some small blue staining on the white area [22]. HDL (100μg protein/g mouse) were injected intravenously (retro-orbital) 1min before reperfusion.

### Microarray analysis

Cardiac miRNA expression was evaluated in 3 different treatment groups (sham, IR and IR+HDL). After 8h of reperfusion mice were re-anaesthetized and hearts (full ventricle to avoid any bias) collected and frozen. Frozen heart tissues were powdered using liquid nitrogen. Total RNA was extracted using miRNeasy Mini kits (Qiagen) and quantified using Qubit 2.0 (Invitrogen). RNA samples were subjected to microarray analysis using GeneChip miRNA 2.0 Arrays (Affymetrix) which measured 3163 mouse probesets (1908 mature miRNA and 1255 pre-miRNA probesets).

### miRNA expression by quantitative PCR

RNA extracted from the *in vivo* experiments, as well as RNA extracted from cardiomyocytes following hypoxia, was reverse transcribed using the miRCURY Locked Nucleic Acid (LNA) Universal Reverse Transcription (RT) microRNA PCR, Polyadenylation and cDNA synthesis kit (Exiqon). cDNA was diluted 80x and assayed in 10μl PCR reactions according to the protocol. The amplification was performed in a LightCycler 480 Real-Time PCR System (Roche) in 96-well plates. The amplification curves were analyzed using the Roche LightCycler software, both for determination of Cp (by the 2nd derivative method) and for melting curve analysis. Primers for mmu-miR-34b (cat n°205086 MIMAT0004581, also valid for rno), mmu-miR-337 (cat n°205184 MIMAT0004644), rno-miR-337 (cat n°2103270 MIMAT0017035) and U6 (cat n° 203907) as housekeeping gene were used (Exiqon).

### Cell culture

Experiments involving animals were approved by the local Animal Ethics Committee (University of Geneva). Neonatal male and female (1 to 2-day-old) Wistar rats were euthanized by decapitation and right ventricles rapidly excised and washed in Hank’s Balanced Salt Solution (HBSS) (Sigma, H9394). Ventricles were then sliced in half and incubated in 50ml of 0.5% trypsin (Sigma, 59418C) at 4ºC overnight with shaking. Trypsin was neutralized with 20ml Dulbecco’s Modified Eagle Medium (DMEM) (Invitrogen 31885023, glucose 1g/L) containing 10% fetal calf serum (FCS) (Biochrom,S0415) and 1% penstrep (Gibco, 15140122) at 37ºC as described in detail previously [23].

### Hypoxia protocol

Before hypoxic exposure, cell medium was replaced by modified Tyrode’s solution (in mM: NaCl 136.9, KCl 2.68, Na_2_HPO_4_.12H_2_O 8.1, KH_2_PO_4_ 1.47, CaCl_2_ 0.9, MgCl_2_ .6H_2_O 0.49; pH 7.2). Cardiomyocytes were placed in an anaerobic hypoxia chamber containing 5% CO_2_ and 95% N_2_ at 37°C for 7h. For normoxic treatment, cells were maintained in DMEM medium at 37°C in a culture incubator with 5% CO_2_. Treated cardiomyocytes were exposed to HDL (400μg protein/ml) during hypoxia.

### Measurement of cell viability

Following hypoxia, cells were loaded with 0.04% trypan blue and cell viability was immediately analysed using a light microscope. The number of viable (unstained) and non-viable (blue stained) cardiomyocytes in 3 random microscopic fields was recorded, with at least 30 cells counted in each field. Cell viability was normalized to the normoxic control.

### Small interfering RNA (siRNA) transfection

Lipofectamine RNAiMAX (Invitrogen) was used to transfect STAT3 siRNA or nonspecific siRNA control into cardiomyocytes according to the recommendations of the manufacturer (Life Technologies). Cells were treated with 50pmol of specific siRNA. After overnight transfection, the complexes were removed, and fresh medium containing 10% FCS was added. STAT3 mRNA expression was analysed using the following procedure: total RNA was extracted (NucleoSpin RNA, Macherey-Nagel) and reverse transcribed using the manufacturers’ protocols. Real-time PCR was performed on a Light-Cycler480II (Roche). Primers to detect STAT3 were (sense) cagcctgtctgcagagttca and (anti-sense) aaggtgatcaggtgcagctc and for housekeeping gene 9S were (sense) ctccggaacaaacggtgaggt and (anti-sense) tccagcttcatcttgccctc.

### miR-34b/miR-337 mimics and miR-34b/miR-337 antimiRs transfection

Cardiomyocytes were seeded in petri dishes. Then, miR-34b and miR-337 mimics (Qiagen) (6nM each) or miR-34b and miR-337 antimiRs (Qiagen) (6nM each) were transfected into cells using HiPerFect Transfection Reagent (Qiagen). After overnight transfection, complexes were removed, and fresh medium containing 10% FCS was added. All stars Negative Control siRNA (12nM) (Qiagen) was used as a negative control. This concentration used was comparable to the one used in other studies [24,25]. The expression of miR-34b and miR-337was evaluated by qPCR and showed that both miRNA concentrations were increased in the presence of mimics (n = 8) (S1 Table). The magnitude of changes observed likely exceeded physiological ranges but the concentrations at which endogenous miRNAs are considered pathophysiologic remain unknown to this day.

### Statistical analysis

Data are expressed as mean ± the standard error of the mean (SEM). Comparisons were performed by two-way ANOVA analysis (miRNA microarray and RNA sequencing analysis) and by Student t-test (one-way paired and unpaired) when applicable. p<0.05 was accepted as significantly different.

## Results

### *In vivo* HDL protects the heart from IR injury via STAT3

**Fig 1:**
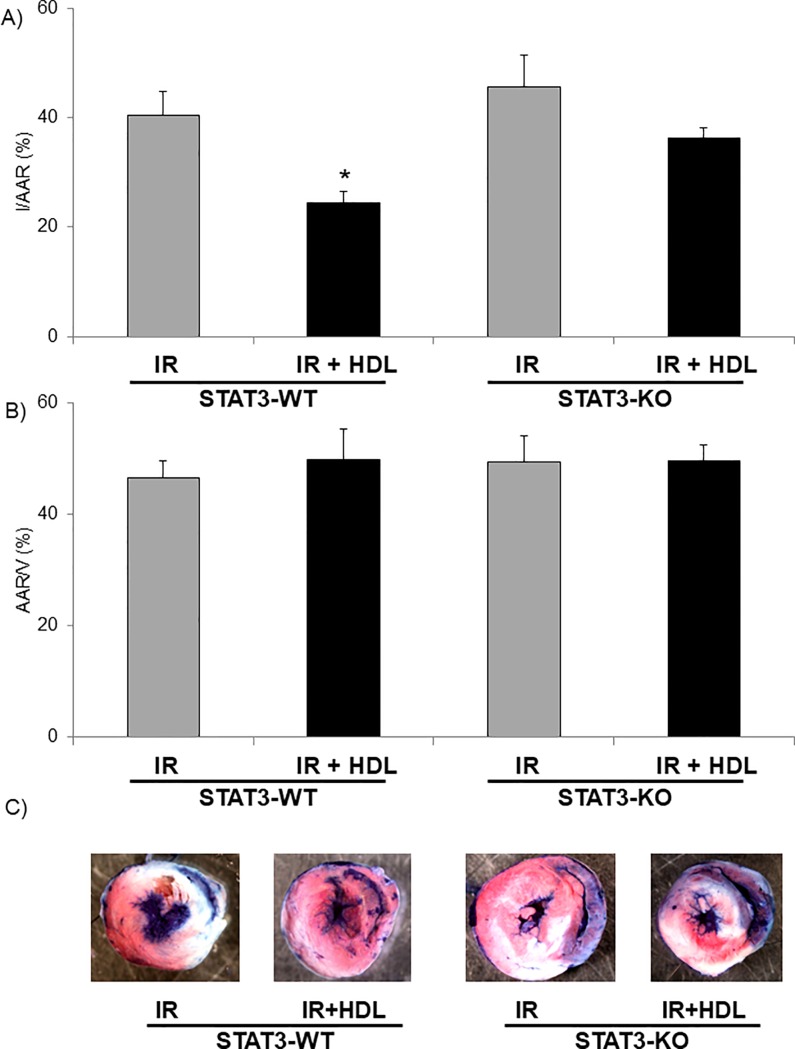
*In vivo* HDL protects the heart from IR injury via STAT3 activation. Mice were submitted to LAD occlusion for 45min and hearts were reperfused for 24h. Mice were injected or not (IR) with HDL (HDL) one minute before reperfusion. A) Quantification of infarct size (I) expressed in % of area at risk (AAR). B) Quantification of AAR per ventricle surface (V). C) Representative images of TTC stained middle heart sections of control or treated mice. Data are mean ± SEM, n = 6–8, *: p<0.05 vs IR.

In order to evaluate the role of STAT3 in the cardioprotective role of HDL *in vivo*, mice were submitted to the IR protocol and infarct size was analysed in STAT3-WT and STAT3-KO mice. After 45min of ischemia followed by 24h reperfusion, HDL significantly reduced IS (p<0.05) in STAT3-WT mice hearts but failed to protect in STAT3-KO mice (Fig 1A and 1C). The AAR/V area was similar between the 4 groups (Fig 1B and 1C).

### *In vivo* HDL regulates specific miRNAs during IR injury

**Fig 2:**
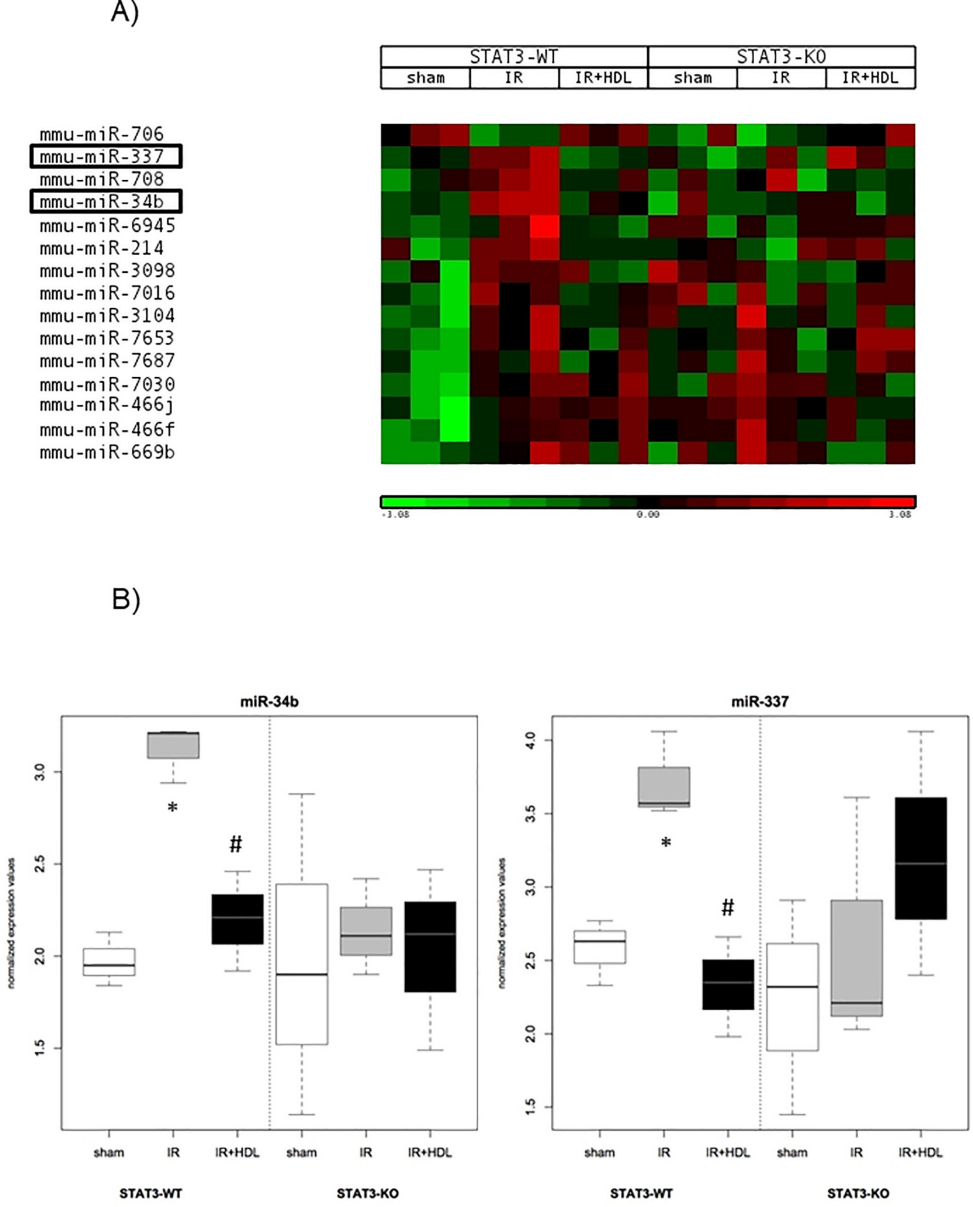
*In vivo* HDL regulates specific miRNAs during IR injury. Mice were sham operated (sham) or submitted to LAD occlusion for 45min and hearts were reperfused for 8h (IR). HDL was injected or not one min before reperfusion. miRNA was isolated from total heart and miRNA expression assessed by microarray analysis after 8h of reperfusion in *in vivo* mouse hearts using GeneChip miRNA 2.0 Arrays. A) miRNA expression significant change (p<0.05) between sham and IR group (Heatmap representation). B) Expression of miR-34b and miR-337 (boxplot representation). n = 3. *: p<0.05 vs sham, #: p<0.05 vs IR.

As a next step, we analysed whether HDL can modulate the expression of miRNA in IR conditioning. The operated mice were divided in 3 groups; sham operated, IR and IR+HDL mice. After 45min of ischemia followed by 8h reperfusion, global miRNA expression was analysed by microarray. In STAT3-WT mice, 15 miRNAs were upregulated and 1 was decreased (p value<0.05 and fold change>2) following IR compared to operated sham mice. Among these potential targets, the expression of miR-34b and miR-337 was significantly changed (p<0.05) when treated with HDL. The expression of miR-34b and miR-337 was increased (fold change 2.22 and 2.21, respectively) in STAT3-WT mice hearts. HDL treatment decreased the expression of both miRNAs (fold change -1.90 and -2.62, respectively). In contrast, the expression of these miRNAs was not modified between conditions in STAT3-KO mice (Fig 2).

**Fig 3:**
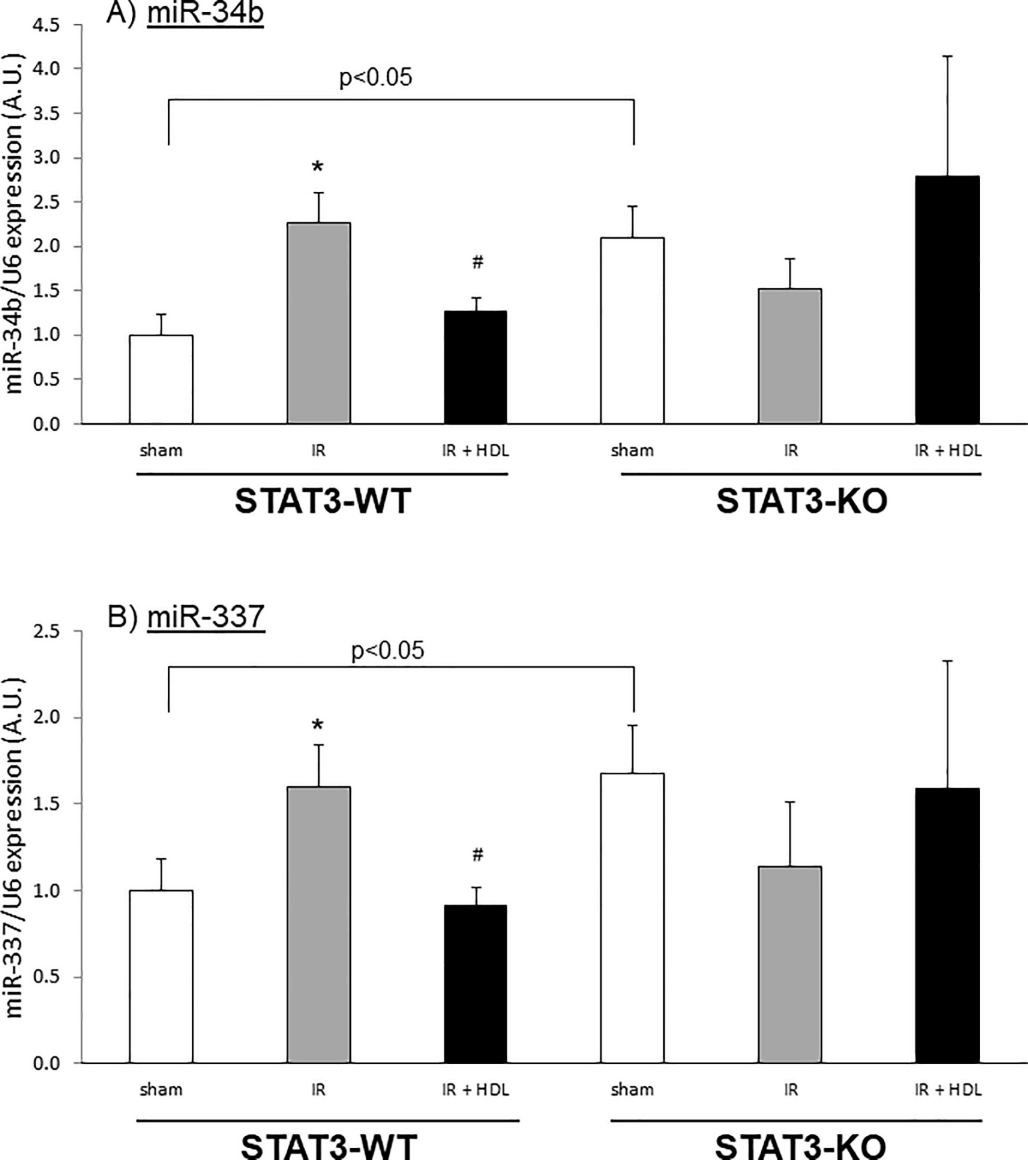
*In vivo* HDL regulates miR-34b and miR-337 during IR injury. Mice were sham operated (sham) or submitted to LAD occlusion for 45min and hearts were reperfused for 8h (IR). HDL was injected or not one min before reperfusion. miRNA was isolated from total heart. A) miR-34b and B) miR-337 expression was measured by qPCR and normalized to U6 expression. Data are mean ± SEM, n = 6–9, *: p<0.05 vs sham, #: p<0.05 vs hypoxia. The difference between sham STAT3-WT and STAT3-KO is significant (p<0.05).

These observations were confirmed by qPCR which showed that the expression of miR-34b (Fig 3A) and miR-337 (Fig 3B) was increased by IR (2.26±0.35 and 1.59±0.24, respectively) and reduced by HDL (1.26±0.15 and 0.91±0.10, respectively) in STAT3-WT mice hearts. In contrast, the absence of STAT3 was associated with a significant increase in miR-34b (p = 0.01) and miR-337 (p = 0.03) already in the sham operated animals (Fig 3A and 3B). Subsequent treatment had no significant impact on miRNA levels (Fig 3A and 3B), as observed by qPCR analysis.

### HDL improves cell survival against hypoxia and regulates specific miRNAs during hypoxia

**Fig 4:**
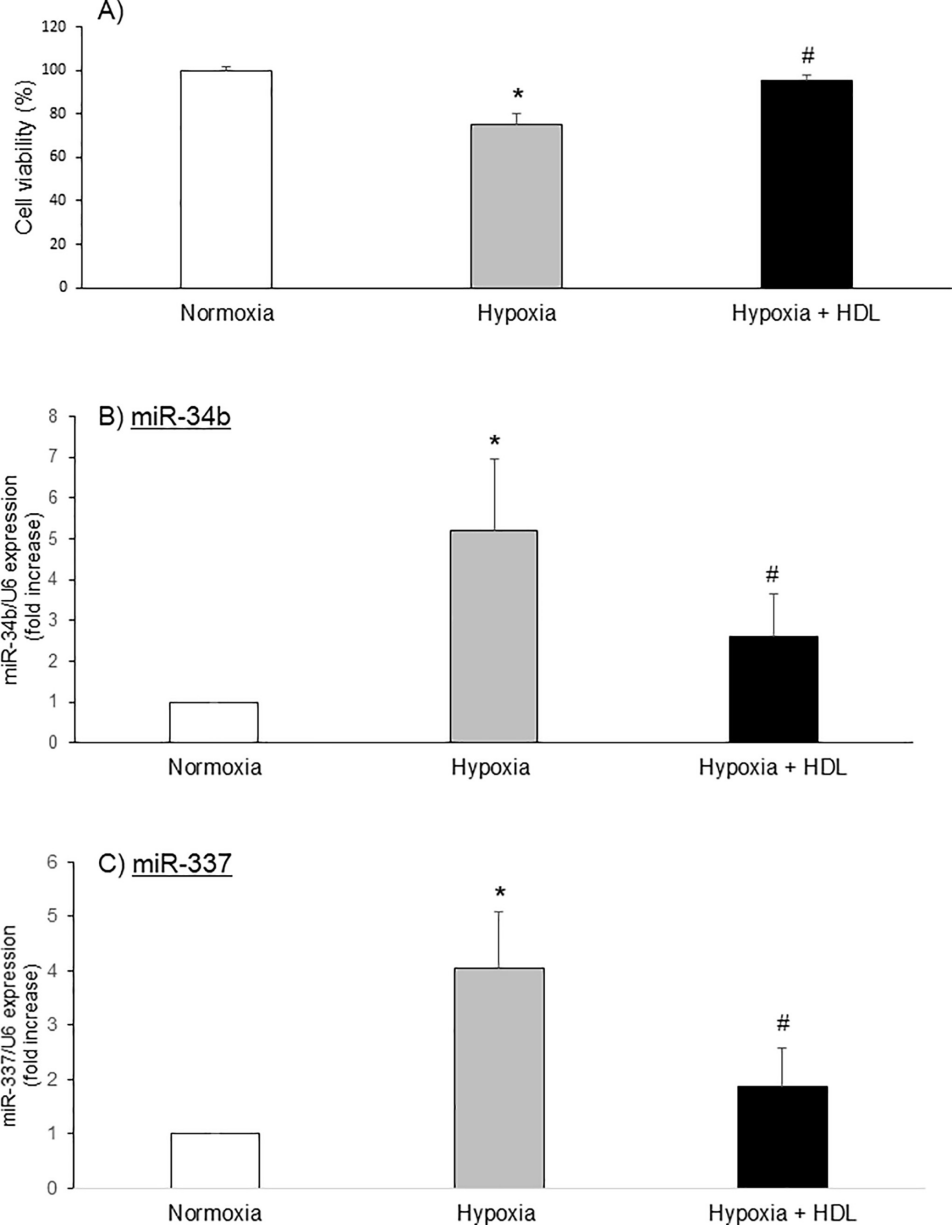
HDL improves cell survival against hypoxia and regulates specific miRNAs during hypoxia. Cardiomyocytes were incubated in Tyrode solution, submitted to 7h hypoxia and treated during hypoxia with HDL. A) Cell viability, B) miR-34b and C) miR-337 expression. Results are expressed in percentage of normoxia. Data are mean ± SEM, n = 8–10, *: p<0.05 vs normoxia, #: p<0.05 vs hypoxia.

In order to understand the role of these miRNAs in cardiomyocyte survival, we used a model of neonatal rat cardiomyocytes. In these cells, the exposure to hypoxia decreased cell survival from 100% (normoxia) to 75.0±5.3%. HDL treatment restored survival to 95.2±2.8% (Fig 4A).

The expression of miR-34b and miR-337 was evaluated in these conditions. Exposure to 7h of hypoxia increased the expression of miR-34b (Fig 4B) and miR-337 (Fig 4C) by approximately 5 and 3 times, respectively. Incubation with HDL significantly reduced the expression of both miRNAs to a level similar to normoxia.

### HDL improves cell survival against hypoxia via STAT3 and miRNA expression

**Fig 5:**
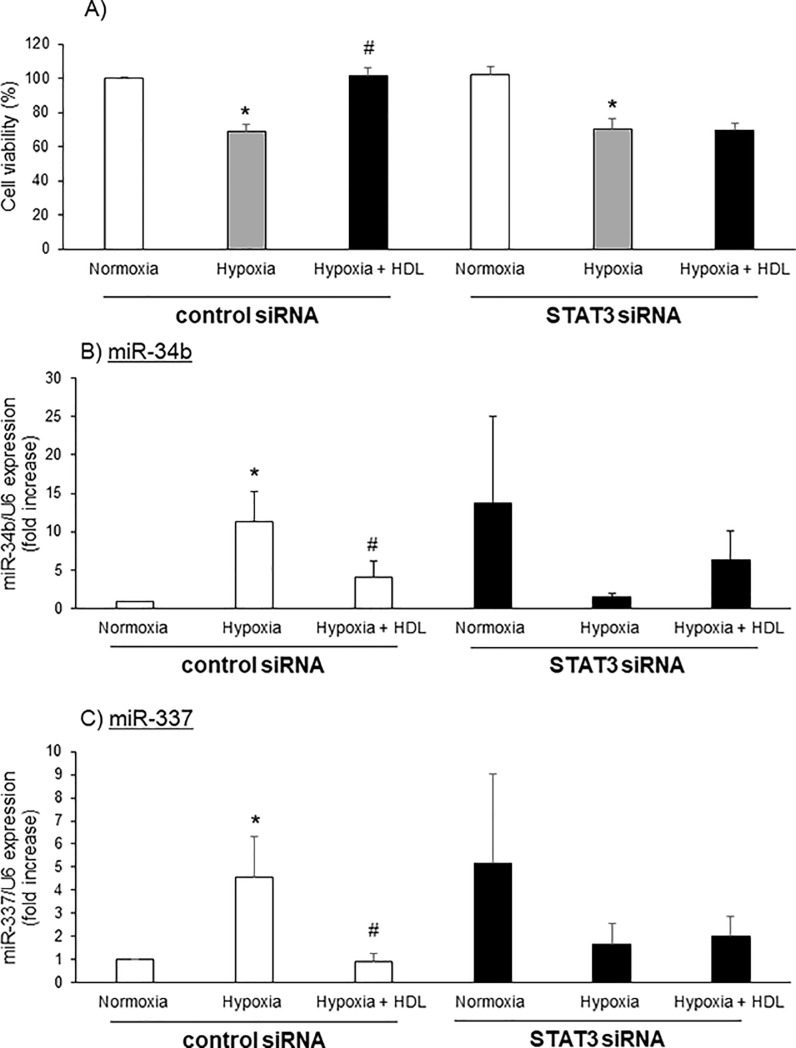
HDL improves cell survival against hypoxia via STAT3 and miRNA expression. Cardiomyocytes transfected with control or STAT3 siRNA were incubated in Tyrode solution, submitted to 7h hypoxia and treated during hypoxia with HDL. A) Cell viability, B) miR-34b and C) miR-337 expression. Results are expressed in percentage of normoxia. Data are mean ± SEM, n = 3–7, *: p<0.05 vs normoxia, #: p<0.05 vs hypoxia.

The specific role of STAT3 was also evaluated using specific STAT3 siRNA. Treatment with STAT3 siRNA showed a 94.0±2.6% reduction in STAT3 mRNA expression (S1 Fig). these cells transfected either with control siRNA or STAT3 siRNA, exposure to 7h of hypoxia decreased cell survival by 30%. Although HDL treatment protected from cell death in control siRNA transfected cells, HDL failed to restore cell survival in cardiomyocytes transfected with STAT3 siRNA (Fig 5A).

Additionally, the expression of miR-34b and miR-337 was altered in the cardiomyocytes transfected with STAT3 siRNA. Correspondingly, HDL lost its effect and failed to modulate the expression of these miRNAs (Fig 5B and 5C).

### miR-34b and miR-337 expression influence cell survival

**Fig 6:**
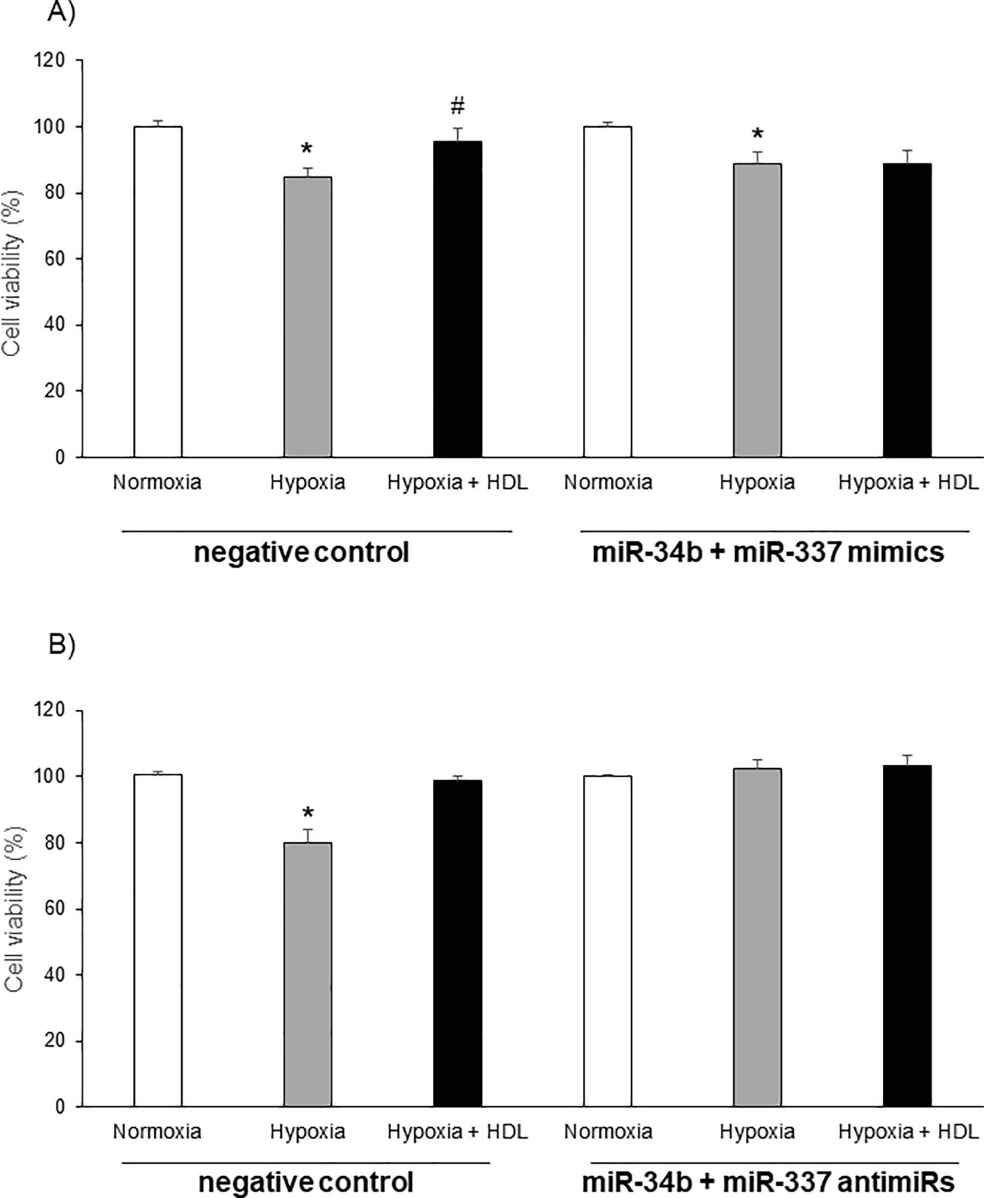
miR-34b and miR-337 expression influences cell survival. Cell viability after 7h of hypoxia in neonatal rat cardiomyocytes treated with negative control, miR-34b and miR-337 mimics (A) or antimiRs (B). Results are expressed in percentage of normoxia. Data are mean ± SEM, n = 5, *: p<0.05 vs normoxia, #: p<0.05 vs hypoxia.

The role of these miRNAs in the hypoxic insult was further evaluated using specific mimics and antimiRs. In order to mimic optimally the situation of hypoxia a mix of both miR-34b and miR-337 mimics or miR-34b and miR-337 antimiRs was used. Exposure to hypoxia of cells transfected with the mix of mimics diminished cell survival by approximately 30%. In these cells, HDL lost its protective capacity (Fig 6A). Conversely cardiomyoytes transfected with a mix of antimiRs were protected against the deleterious insult of hypoxia independently of the HDL treatment (Fig 6B).

## Discussion

The main findings in our study strongly suggest that HDL confers protection against IR via the can modulation of the expression of miRNAs. In addition, our data show that this modulation by HDL is mediated via the activation of the pro-survival protein STAT3 whose role in HDL-mediated protection has previously been demonstrated in details [6–8].

HDL is a major negative predictor for cardiovascular events. New evidence has demonstrated that HDL has more widespread beneficial effects beyond its role in reverse cholesterol transport [2]. These beneficial effects appear to act via a direct interaction with cells. Although the mechanisms by which HDL can affect cell function remain to be fully elucidated, several studies showed that HDL is capable of influencing a number of intracellular signalling cascades involved in multiple cell survival processes. Little is known concerning the direct effect of HDL in the heart. A few studies, including our own, demonstrated that HDL protects the heart against stress [4–7,21]. However, the precise mechanisms of action have not been fully delineated, underlining the need to expand our understanding on the interaction between HDL and the heart. In our present study, we used human HDL in rodent models. The use of human HDL in rodent models is commonly reported in the literature and has been experimented without any evidence of problems [4,26–30]. Similarly, several studies have used transgenic mice expressing human apoA1, which is the major protein content in HDL particle [31–33]. In order to ensure that HDL derived from different species act in a similar manner in our model, we performed *in vitro* experiments and showed that both human and murine HDL exerted similar effects on cell death (S2 Fig) as well as on ERK1/2 phosphorylation (S3 Fig) in endothelial cells.

In this study, we evaluated HDL impact on miRNA expression under conditions of IR. The use of the microarray approach allows an objective analysis of the expression of miRNA modulated by IR and HDL treatment *in vivo*. It revealed miRNAs whose expression was modulated by IR compared to sham-treated mice. Among these miRNAs, only a few were affected by HDL treatment compared to those in IR. The analysis highlighted the expression of 2 miRNAs: miR-34b and miR-337. These experiments were extended *in vitro*. Similar to the data obtained *in vivo*, in cultured cardiomyocytes hypoxia induced an increase in miR-34b and miR-337 expression. Experiments using mimics and anti-miRNAs strongly support a role in cell survival under ischemia. Our data demonstrate that a decrease in the expression of these miRNA induced by HDL treatment could improve cardiac cell survival. The potential effect of HDL on miRNAs was only investigated in IR protocol as a particle able to trigger cardioprotective effects. It would also be of interest to explore the regulation of miRNAs following a treatment of HDL in hearts not subjected to IR. In endothelial cells, Tabet et al [34] demonstrated that HDL altered endothelial cell miRNA levels with significant differential miRNA following a treatment with native HDL (3 in total; 1 down: miR-339-5p; 2 up: miR-223 and miR-577).

Our previous publications underlined the role of STAT3 in the protective effect of HDL [6,7]. We also demonstrated that, in the heart, HDL induces the serine phosphorylation of STAT3 after 5 min of stimulation in vitro, ex vivo and in vivo [8,9,21]. Sekine et al. [35] and Feuerborn et al. [36] confirmed a similar effect in prostate cancer DU145 cells and in RAW264.7 murine macrophages, respectively.

In the present study, we used specific cardiomyocyte STAT3 knockout mice to confirm *in vivo* the role of STAT3 in the HDL-induced cardioprotection cardioprotective effects, therefore supporting our previous work conducted in an isolated heart model [6]. Furthermore, we now demonstrate that STAT3 plays a role in the modulation of the expression of miR-34b and miR-337 *in vivo*. These results were confirmed *in vitro* where STAT3 siRNA transfected cardiomyocytes were not protected by HDL treatment. Interestingly, in sham treated animals, absence of STAT3 was associated with a significant increase in both miRNAs (Fig 3A and 3B).

Although the precise role of these specific miRNAs has not been investigated in myocardial IR, Bernardo and colleagues investigated the role of the miR-34 family in the heart submitted to stress [37]. Under conditions of myocardial infarction, the miR-34 family is upregulated whereas inhibition improves heart function. Unfortunately, global inhibition of miR-34a, b and c was used, thus not allowing the evaluation of the specific role of miR-34b in this response [37]. According to our data, miR-34b could play an important role in the effect of miR-34 family. In cardiac studies, miR-34b is associated with congenital disease [38] and is dysregulated in patients with diabetic ischemic heart failure [39]. miR-337 is increased in plasma from coronary artery disease patients compared to control subjects [40] and its expression is modulated in the regenerative process of neonatal ventricular cells [41]. Unfortunately, its role in these different pathologies was not investigated. It should be noted that most of the knowledge on these miRNAs comes from cancer studies. In tumoral cells, miR-337 suppresses cell proliferation, migration, and invasion in human pancreatic cancer cell lines [42] and senescence in human colorectal cancer cells [43]. miR-34b is downregulated in prostate cancer cells, in colonic tumours, gastric carcinogenesis, pancreatic cancer metastasis [44–47] and is correlated with higher lymph node metastasis [48]. Additionally induction of its expression induces cell death in ovarian cancer cells [49]. While an increase of miR-337 expression induced cell death, a decrease in miR-34b expression seems to improve cell survival. This is therefore in agreement with our data. Likewise, miR-34b is upregulated in neuronal PC12 cells exposed to sevoflurane preconditioning [50], suggesting that an increase in miR-34b expression could be protective in neuronal cells. Comparing these studies with our own is difficult, given the different cell type and different pathophysiological conditions.

Thus, our data suggest a new role for these miRNAs in the heart, a role that HDL can beneficially influence by modulating their expression.

There is a growing body of evidence demonstrating that miRNAs are closely associated with the STAT3 signalling pathway. Modulation of STAT3 expression essentially influenced cell survival. Although most of these data have been obtained from cancer cells [14], a few articles described the link between miRNA and STAT3 in cardiac tissue. Among these articles, only one study shows that reduction of STAT3 levels can modulate miRNA expression. More precisely, knock-down of STAT3 increased miR-199a expression and subsequent impairment of the ubiquitin-proteasome system [15]. In the other studies, STAT3 was targeted by miR-17, miR-21 in cardiomyocytes [51,52] and miR-351 in endothelial cells [53]. The reduction of STAT3 protein expression impaired cell survival. In the present study, we therefore highlight a novel aspect on the role of STAT3 in HDL-induced cardioprotection by demonstrating the influence of HDL-activated STAT3 on the expression of miR-34b and miR-337.

**Fig 7:**
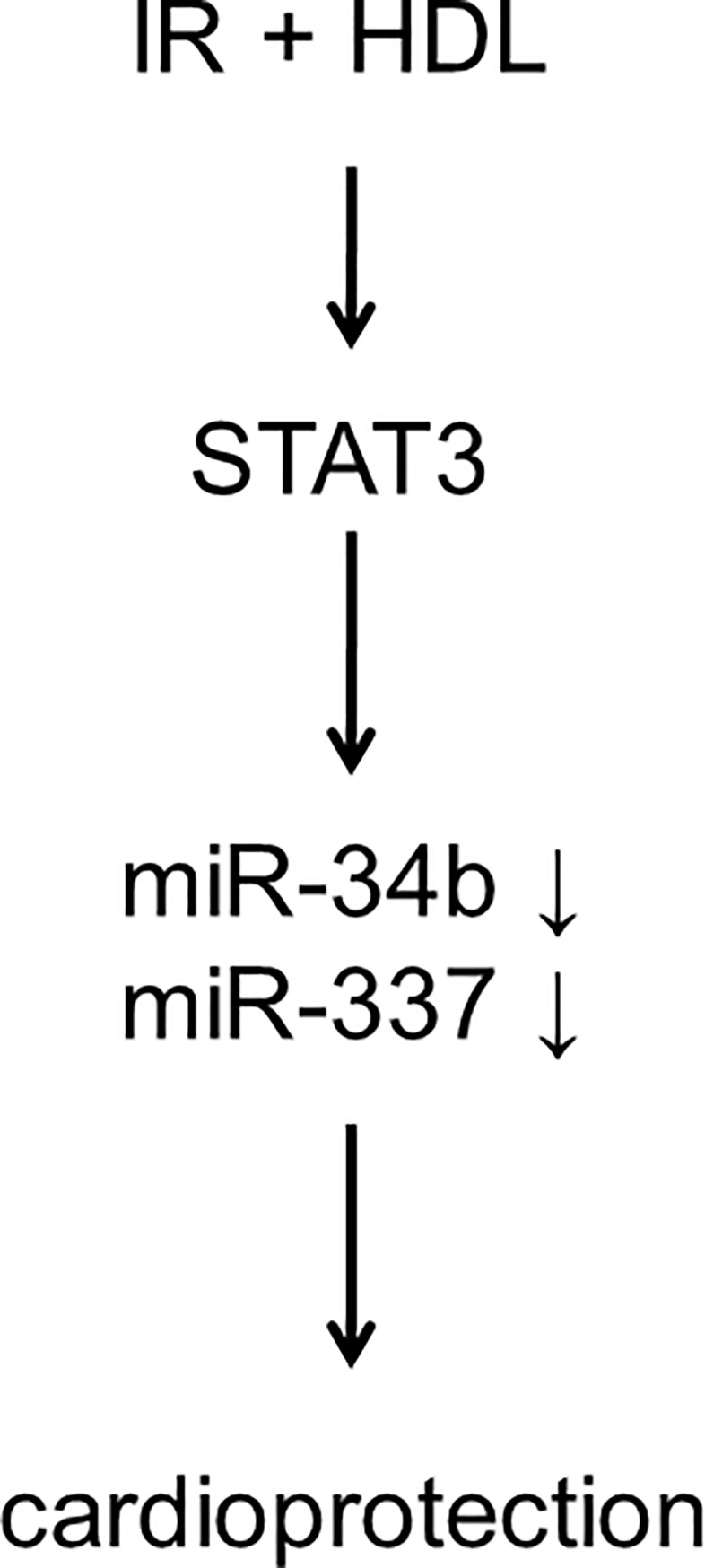
Proposed mechanism of HDL cardioprotection against IR injury occurring via a STAT3-dependent decrease of miR-34b and miR-337 expression.

Our data identify a novel pathway by which HDL can protect the heart from IR injury with the modulation of miR-337 and miR-34b expression as critical downstream target of STAT3 (see Fig 7). It is, to our knowledge, the first study to demonstrate a role for HDL in modulating miRNA expression following IR. This novel aspect of HDL action in the heart expands our understanding in the cardioprotective effects of HDL. Combination of these protective influences underlines the therapeutic potential of HDL in the heart.

## Supporting information

S1 Table (DOCX)

S1 Fig (DOCX)

S2 Fig (DOCX)

S3 Fig (DOCX)

## References

[1] Barter PJ, Rye KA. High density lipoproteins and coronary heart disease. *Atherosclerosis*. 1996;121(1):1–12. 8678914 10.1016/0021-9150(95)05675-0

[2] Nofer JR, Kehrel B, Fobker M, Levkau B, Assmann G, von Eckardstein A. HDL and arteriosclerosis: beyond reverse cholesterol transport. *Atherosclerosis*. 2002;161(1):1–16. 11882312 10.1016/s0021-9150(01)00651-7

[3] Calabresi L, Rossoni G, Gomaraschi M, Sisto F, Berti F, Franceschini G. High-density lipoproteins protect isolated rat hearts from ischemia-reperfusion injury by reducing cardiac tumor necrosis factor-alpha content and enhancing prostaglandin release. *Circulation research*. 2003;92(3):330–337. 12595346 10.1161/01.res.0000054201.60308.1a

[4] Theilmeier G, Schmidt C, Herrmann J, Keul P, Schafers M, Herrgott I et al High-density lipoproteins and their constituent, sphingosine-1-phosphate, directly protect the heart against ischemia/reperfusion injury in vivo via the S1P3 lysophospholipid receptor. *Circulation*. 2006;114(13):1403–1409. 16982942 10.1161/CIRCULATIONAHA.105.607135

[5] Tao R, Hoover HE, Honbo N, Kalinowski M, Alano CC, Karliner JSet al. High-density lipoprotein determines adult mouse cardiomyocyte fate after hypoxia-reoxygenation through lipoprotein-associated sphingosine 1-phosphate. *American journal of physiology Heart and circulatory physiology*. 2010;298(3):H1022–1028. 10.1152/ajpheart.00902.2009 20061542 PMC2838562

[6] Frias MA, Pedretti S, Hacking D, Somers S, Lacerda L, Opie LH et al HDL protects against ischemia reperfusion injury by preserving mitochondrial integrity. *Atherosclerosis*. 2013;228(1):110–116. 10.1016/j.atherosclerosis.2013.02.003 23497785

[7] Frias MA, Lang U, Gerber-Wicht C, James RW. Native and reconstituted HDL protect cardiomyocytes from doxorubicin-induced apoptosis. *Cardiovascular research*. 2010;85(1):118–126. 10.1093/cvr/cvp289 19700468

[8] Frias MA, James RW, Gerber-Wicht C, Lang U. Native and reconstituted HDL activate Stat3 in ventricular cardiomyocytes via ERK1/2: role of sphingosine-1-phosphate. *Cardiovascular research*. 2009;82(2):313–323. 10.1093/cvr/cvp024 19151362

[9] Frias MA, Lecour S, James RW, Pedretti S. High density lipoprotein/sphingosine-1-phosphate-induced cardioprotection: Role of STAT3 as part of the SAFE pathway. Jak-Stat. 2012;1(2):92–100. 10.4161/jkst.19754 24058758 PMC3670301

[10] D'Alessandra Y, Pompilio G, Capogrossi MC. MicroRNAs and myocardial infarction. Current opinion in cardiology. 2012;27(3):228–235. 10.1097/HCO.0b013e3283522052 22476028

[11] Dong S, Cheng Y, Yang J, Li J, Liu X, Wang X et al MicroRNA expression signature and the role of microRNA-21 in the early phase of acute myocardial infarction. *The Journal of biological chemistry*. 2009;284(43):29514–29525. 10.1074/jbc.M109.027896 19706597 PMC2785585

[12] Ren XP, Wu J, Wang X, Sartor MA, Qian J, Jones K et al MicroRNA-320 is involved in the regulation of cardiac ischemia/reperfusion injury by targeting heat-shock protein 20. *Circulation*. 2009;119(17):2357–2366. 10.1161/CIRCULATIONAHA.108.814145 19380620 PMC2746735

[13] Varga ZV, Zvara A, Farago N, Kocsis GF, Pipicz M, Gaspar R et al MicroRNAs associated with ischemia-reperfusion injury and cardioprotection by ischemic pre- and postconditioning: protectomiRs. *American journal of physiology Heart and circulatory physiology*. 2014;307(2):H216–227. 10.1152/ajpheart.00812.2013 24858849

[14] Cao Q, Li YY, He WF, Zhang ZZ, Zhou Q, Liu X et al Interplay between microRNAs and the STAT3 signaling pathway in human cancers. *Physiological genomics*. 2013;45(24):1206–1214. 10.1152/physiolgenomics.00122.2013 24192393

[15] Haghikia A, Missol-Kolka E, Tsikas D, Venturini L, Brundiers S, Castoldi M et al Signal transducer and activator of transcription 3-mediated regulation of miR-199a-5p links cardiomyocyte and endothelial cell function in the heart: a key role for ubiquitin-conjugating enzymes. *European heart journal*. 2011;32(10):1287–1297. 10.1093/eurheartj/ehq369 20965886

[16] Vickers KC, Palmisano BT, Shoucri BM, Shamburek RD, Remaley AT. MicroRNAs are transported in plasma and delivered to recipient cells by high-density lipoproteins. *Nature cell biology*. 2011;13(4):423–433. 10.1038/ncb2210 21423178 PMC3074610

[17] Vickers KC, Landstreet SR, Levin MG, Shoucri BM, Toth CL, Taylor RC et al MicroRNA-223 coordinates cholesterol homeostasis. *Proceedings of the National Academy of Sciences of the United States of America*. 2014;111(40):14518–14523. 10.1073/pnas.1215767111 25246565 PMC4210029

[18] Niculescu LS, Simionescu N, Sanda GM, Carnuta MG, Stancu CS, Popescu AC et al MiR-486 and miR-92a Identified in Circulating HDL Discriminate between Stable and Vulnerable Coronary Artery Disease Patients. *PloS one*. 2015;10(10):e0140958 10.1371/journal.pone.0140958 26485305 PMC4617647

[19] Smith RM, Suleman N, Lacerda L, Opie LH, Akira S, Chien KR et al Genetic depletion of cardiac myocyte STAT-3 abolishes classical preconditioning. *Cardiovascular research*. 2004;63(4):611–616. 10.1016/j.cardiores.2004.06.019 15306216

[20] James RW, Proudfoot A, Pometta D. Immunoaffinity fractionation of high-density lipoprotein subclasses 2 and 3 using anti-apolipoprotein A-I and A-II immunosorbent gels. *Biochimica et biophysica acta*. 1989;1002(3):292–301. 10.1016/0005-2760(89)90343-3 2469471

[21] Brulhart-Meynet MC, Braunersreuther V, Brinck J, Montecucco F, Prost JC, Thomas A, et al Improving reconstituted HDL composition for efficient post-ischemic reduction of ischemia reperfusion injury. *PloS one*. 2015;10(3):e0119664 10.1371/journal.pone.0119664 25781943 PMC4362758

[22] Redfors B, Shao Y, Omerovic E. Myocardial infarct size and area at risk assessment in mice. *Exp Clin Cardiol*. 2012;17(4):268–272. 23592952 PMC3627291

[23] Frias MA, Somers S, Gerber-Wicht C, Opie LH, Lecour S, Lang U. The PGE2-Stat3 interaction in doxorubicin-induced myocardial apoptosis. *Cardiovascular research*. 2008;80(1):69–77. 10.1093/cvr/cvn171 18567640

[24] Clark AL, Naya FJ. MicroRNAs in the Myocyte Enhancer Factor 2 (MEF2)-regulated Gtl2-Dio3 Noncoding RNA Locus Promote Cardiomyocyte Proliferation by Targeting the Transcriptional Coactivator Cited2. *The Journal of biological chemistry*. 2015;290(38):23162–23172. 10.1074/jbc.M115.672659 26240138 PMC4645594

[25] Zhang ZW, Li H, Chen SS, Li Y, Cui ZY, Ma J. MicroRNA-122 regulates caspase-8 and promotes the apoptosis of mouse cardiomyocytes. *Brazilian journal of medical and biological research = Revista brasileira de pesquisas medicas e biologicas / Sociedade Brasileira de Biofisica* [et al]. 2017;50(2):e5760. 10.1590/1414-431X20165760 PMC5390529 28177059

[26] Levkau B, Hermann S, Theilmeier G, van der Giet M, Chun J, Schober O et al High-density lipoprotein stimulates myocardial perfusion in vivo. *Circulation*. 2004;110(21):3355–3359. 10.1161/01.CIR.0000147827.43912.AE 15545521

[27] Mishra M, Muthuramu I, Aboumsallem JP, Kempen H, De Geest B. Reconstituted HDL (Milano) Treatment Efficaciously Reverses Heart Failure with Preserved Ejection Fraction in Mice. *International journal of molecular sciences*. 2018;19(11). 10.3390/ijms19113399 PMC6274776 30380754

[28] Xu B, Gillard BK, Gotto AM Jr., Rosales C, Pownall HJ. ABCA1-Derived Nascent High-Density Lipoprotein-Apolipoprotein AI and Lipids Metabolically Segregate. *Arteriosclerosis*, *thrombosis*, *and vascular biology*. 2017;37(12):2260–2270. 10.1161/ATVBAHA.117.310290 29074589 PMC5831354

[29] Thacker SG, Zarzour A, Chen Y, Alcicek MS, Freeman LA, Sviridov DO et al High-density lipoprotein reduces inflammation from cholesterol crystals by inhibiting inflammasome activation. *Immunology*. 2016;149(3):306–319. 10.1111/imm.12638 27329564 PMC5046053

[30] Bursill CA, Castro ML, Beattie DT, Nakhla S, van der Vorst E, Heather AK et al High-density lipoproteins suppress chemokines and chemokine receptors in vitro and in vivo. *Arteriosclerosis*, *thrombosis*, *and vascular biology*. 2010;30(9):1773–1778. 10.1161/ATVBAHA.110.211342 20702809

[31] Durham KK, Chathely KM, Mak KC, Momen A, Thomas CT, Zhao YY et al HDL protects against doxorubicin-induced cardiotoxicity in a scavenger receptor class B type 1-, PI3K-, and Akt-dependent manner. *American journal of physiology Heart and circulatory physiology*. 2018;314(1):H31–H44. 10.1152/ajpheart.00521.2016 28986362

[32] Tiniakou I, Kanaki Z, Georgopoulos S, Chroni A, Van Eck M, Fotakis P et al Natural human apoA-I mutations L141RPisa and L159RFIN alter HDL structure and functionality and promote atherosclerosis development in mice. *Atherosclerosis*. 2015;243(1):77–85. 10.1016/j.atherosclerosis.2015.08.028 26363436

[33] Hewing B, Parathath S, Barrett T, Chung WK, Astudillo YM, Hamada T et al Effects of native and myeloperoxidase-modified apolipoprotein a-I on reverse cholesterol transport and atherosclerosis in mice. *Arteriosclerosis*, *thrombosis*, *and vascular biology*. 2014;34(4):779–789. 10.1161/ATVBAHA.113.303044 24407029 PMC3966977

[34] Tabet F, Vickers KC, Cuesta Torres LF, Wiese CB, Shoucri BM et al HDL-transferred microRNA-223 regulates ICAM-1 expression in endothelial cells. *Nature communications*. 2014;5:3292 10.1038/ncomms4292 24576947 PMC4189962

[35] Sekine Y, Suzuki K, Remaley AT. HDL and sphingosine-1-phosphate activate stat3 in prostate cancer DU145 cells via ERK1/2 and S1P receptors, and promote cell migration and invasion. *The Prostate*. 2011;71(7):690–699. 10.1002/pros.21285 20979115 PMC4159087

[36] Feuerborn R, Becker S, Poti F, Nagel P, Brodde M, Schmidt H et al High density lipoprotein (HDL)-associated sphingosine 1-phosphate (S1P) inhibits macrophage apoptosis by stimulating STAT3 activity and survivin expression. *Atherosclerosis*. 2017;257:29–37. 10.1016/j.atherosclerosis.2016.12.009 28038379

[37] Bernardo BC, Gao XM, Winbanks CE, Boey EJ, Tham YK, Kiriazis H et al Therapeutic inhibition of the miR-34 family attenuates pathological cardiac remodeling and improves heart function. *Proceedings of the National Academy of Sciences of the United States of America*. 2012;109(43):17615–17620. 10.1073/pnas.1206432109 23047694 PMC3491509

[38] Liu YM, Wang Y, Peng W, Wu Z, Wang XH, Wang ML et al Single-nucleotide polymorphism of the pri-miR-34b/c gene is not associated with susceptibility to congenital heart disease in the Han Chinese population. *Genetics and molecular research*: *GMR*. 2013;12(3):2937–2944. 10.4238/2013.August.12.9 24065649

[39] Greco S, Fasanaro P, Castelvecchio S, D'Alessandra Y, Arcelli D, Di Donato M et al MicroRNA dysregulation in diabetic ischemic heart failure patients. *Diabetes*. 2012;61(6):1633–1641. 10.2337/db11-0952 22427379 PMC3357263

[40] D'Alessandra Y, Carena MC, Spazzafumo L, Martinelli F, Bassetti B, Devanna P et al Diagnostic potential of plasmatic MicroRNA signatures in stable and unstable angina. PloS one. 2013;8(11):e80345 10.1371/journal.pone.0080345 24260372 PMC3829878

[41] Liu HL, Zhu JG, Liu YQ, Fan ZG, Zhu C, Qian LM. Identification of the microRNA expression profile in the regenerative neonatal mouse heart by deep sequencing. *Cell biochemistry and biophysics*. 2014;70(1):635–642. 10.1007/s12013-014-9967-7 24756729

[42] Zhang R, Leng H, Huang J, Du Y, Wang Y, Zang W et al miR-337 regulates the proliferation and invasion in pancreatic ductal adenocarcinoma by targeting HOXB7. Diagnostic pathology. 2014;9:171 10.1186/s13000-014-0171-2 25183455 PMC4164712

[43] Kim SY, Lee YH, Bae YS. MiR-186, miR-216b, miR-337-3p, and miR-760 cooperatively induce cellular senescence by targeting alpha subunit of protein kinase CKII in human colorectal cancer cells. *Biochemical and biophysical research communications*. 2012;429(3–4):173–179. 10.1016/j.bbrc.2012.10.117 23137536

[44] Forno I, Ferrero S, Russo MV, Gazzano G, Giangiobbe S, Montanari E et al Deregulation of MiR-34b/Sox2 Predicts Prostate Cancer Progression. *PloS one*. 2015;10(6):e0130060 10.1371/journal.pone.0130060 26107383 PMC4479381

[45] Hiyoshi Y, Schetter AJ, Okayama H, Inamura K, Anami K, Nguyen GH et al Increased microRNA-34b and -34c predominantly expressed in stromal tissues is associated with poor prognosis in human colon cancer. *PloS one*. 2015;10(4):e0124899 10.1371/journal.pone.0124899 25894979 PMC4404052

[46] Wang AM, Huang TT, Hsu KW, Huang KH, Fang WL, Yang MH et al Yin Yang 1 is a target of microRNA-34 family and contributes to gastric carcinogenesis. *Oncotarget*. 2014;5(13):5002–5016. 10.18632/oncotarget.2073 24970812 PMC4148117

[47] Liu C, Cheng H, Shi S, Cui X, Yang J, Chen L et al MicroRNA-34b inhibits pancreatic cancer metastasis through repressing Smad3. *Current molecular medicine*. 2013;13(4):467–478. 23305226 10.2174/1566524011313040001

[48] Wang LG, Ni Y, Su BH, Mu XR, Shen HC, Du JJ. MicroRNA-34b functions as a tumor suppressor and acts as a nodal point in the feedback loop with Met. *International journal of oncology*. 2013;42(3):957–962. 10.3892/ijo.2013.1767 23314612

[49] Xie YL, Yang YJ, Tang C, Sheng HJ, Jiang Y, Han K et al Estrogen combined with progesterone decreases cell proliferation and inhibits the expression of Bcl-2 via microRNA let-7a and miR-34b in ovarian cancer cells. Clinical & translational oncology: official publication of the Federation of Spanish Oncology Societies and of the National Cancer Institute of Mexico. 2014;16(10):898–905. 10.1007/s12094-014-1166-x 24643702

[50] Sun Y, Li Y, Liu L, Wang Y, Xia Y, Zhang L et al Identification of miRNAs Involved in the Protective Effect of Sevoflurane Preconditioning Against Hypoxic Injury in PC12 Cells. *Cellular and molecular neurobiology*. 2015;35(8):1117–1125. 10.1007/s10571-015-0205-7 25982511 PMC11488055

[51] Du W, Pan Z, Chen X, Wang L, Zhang Y, Li S et al By targeting Stat3 microRNA-17-5p promotes cardiomyocyte apoptosis in response to ischemia followed by reperfusion. Cellular physiology and biochemistry: international journal of experimental cellular physiology, biochemistry, and pharmacology. 2014;34(3):955–965. 10.1159/000366312 25200830

[52] Haider KH, Idris NM, Kim HW, Ahmed RP, Shujia J, Ashraf M. MicroRNA-21 is a key determinant in IL-11/Stat3 anti-apoptotic signalling pathway in preconditioning of skeletal myoblasts. *Cardiovascular research*. 2010;88(1):168–178. 10.1093/cvr/cvq151 20498256 PMC2936124

[53] Zhang Y, Liu Y, Zhang H, Wang M, Zhang J. Mmu-miR-351 attenuates the survival of cardiac arterial endothelial cells through targeting STAT3 in the atherosclerotic mice. *Biochemical and biophysical research communications*. 2015;468(1–2):300–305. 10.1016/j.bbrc.2015.10.108 26505789

