# Supplementary material for: HDL protects against myocardial ischemia reperfusion injury via miR-34b and miR-337 expression which requires STAT3
Source: PLoS One. 2019 Jun 20;14(6):e0218432. doi: 10.1371/journal.pone.0218432 (PMC6586303; doi:10.1371/journal.pone.0218432)
Supplement: S1 Table — (DOCX) [file pone.0218432.s001.docx]

**Supporting Information**

S1Table

| **Condition** | **analysis** | **median** | **1st quartile** | **3rd quartile** |
| --- | --- | --- | --- | --- |
| negative control | miR34b/U6 | 1.0 | 1.0 | 1.0 |
| miR-34b + miR-337 mimics | miR34b/U6 | 1758.3 | 157.7 | 3065.8 |
| negative control | miR337/U6 | 1.0 | 1.0 | 1.0 |
| miR-34b + miR-337 mimics | miR337/U6 | 359.6 | 49.6 | 6248.3 |

**S1 Table: miRNA expression**

miRNA expression under normoxia in neonatal rat cardiomyocytes treated with negative control or miR-34b and miR-337 mimics (n=8).
