# Supplementary material for: HDL protects against myocardial ischemia reperfusion injury via miR-34b and miR-337 expression which requires STAT3
Source: PLoS One. 2019 Jun 20;14(6):e0218432. doi: 10.1371/journal.pone.0218432 (PMC6586303; doi:10.1371/journal.pone.0218432)
Supplement: S1 Fig — (DOCX) [file pone.0218432.s002.docx]

**Supporting Information**

S1 Fig


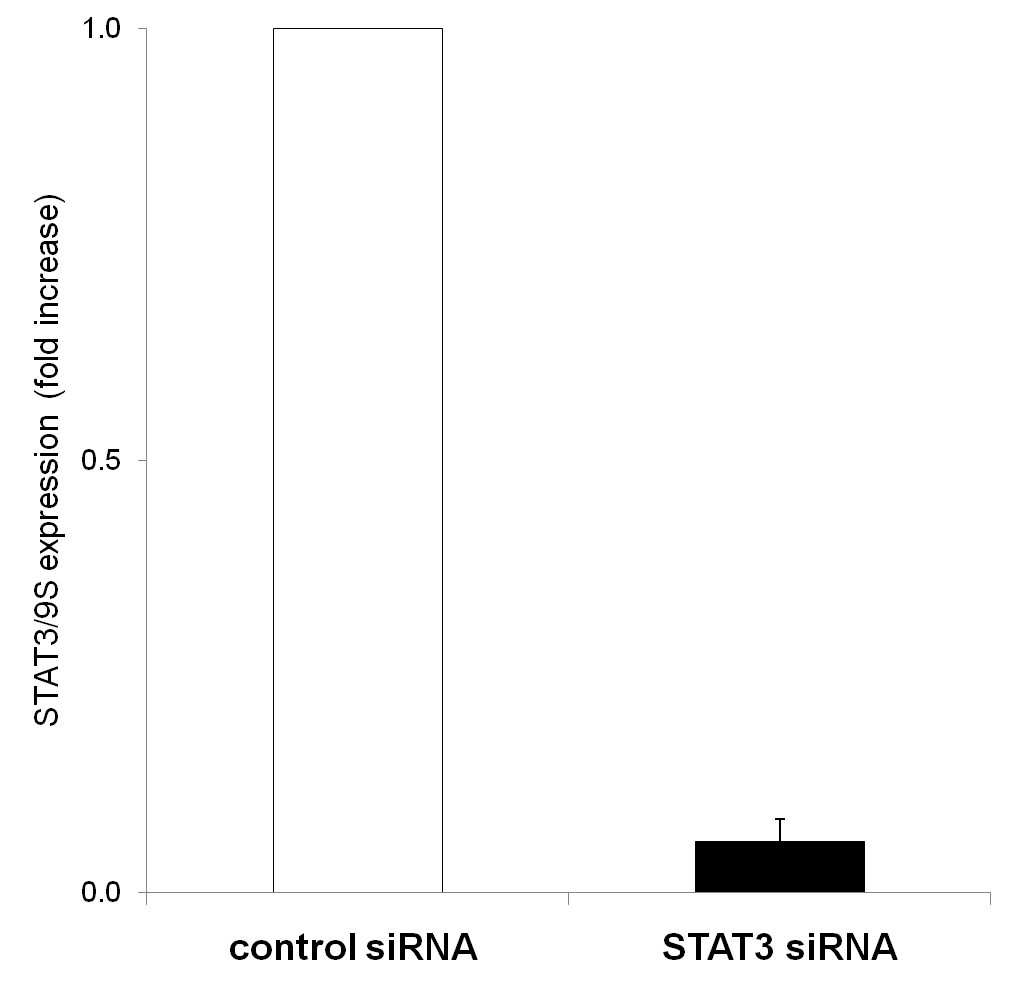


**S1 Fig: STAT3 siRNA treatment reduces STAT3 mRNA level.**

STAT3 mRNA level in neonatal rat cardiomyocytes treated with control or STAT3 siRNA; mean ± SEM, n=8, *: p<0.05 vs control siRNA.
