# Supplementary material for: HDL protects against myocardial ischemia reperfusion injury via miR-34b and miR-337 expression which requires STAT3
Source: PLoS One. 2019 Jun 20;14(6):e0218432. doi: 10.1371/journal.pone.0218432 (PMC6586303; doi:10.1371/journal.pone.0218432)
Supplement: S2 Fig — (DOCX) [file pone.0218432.s003.docx]

**Supporting Information**

S2 Fig

**
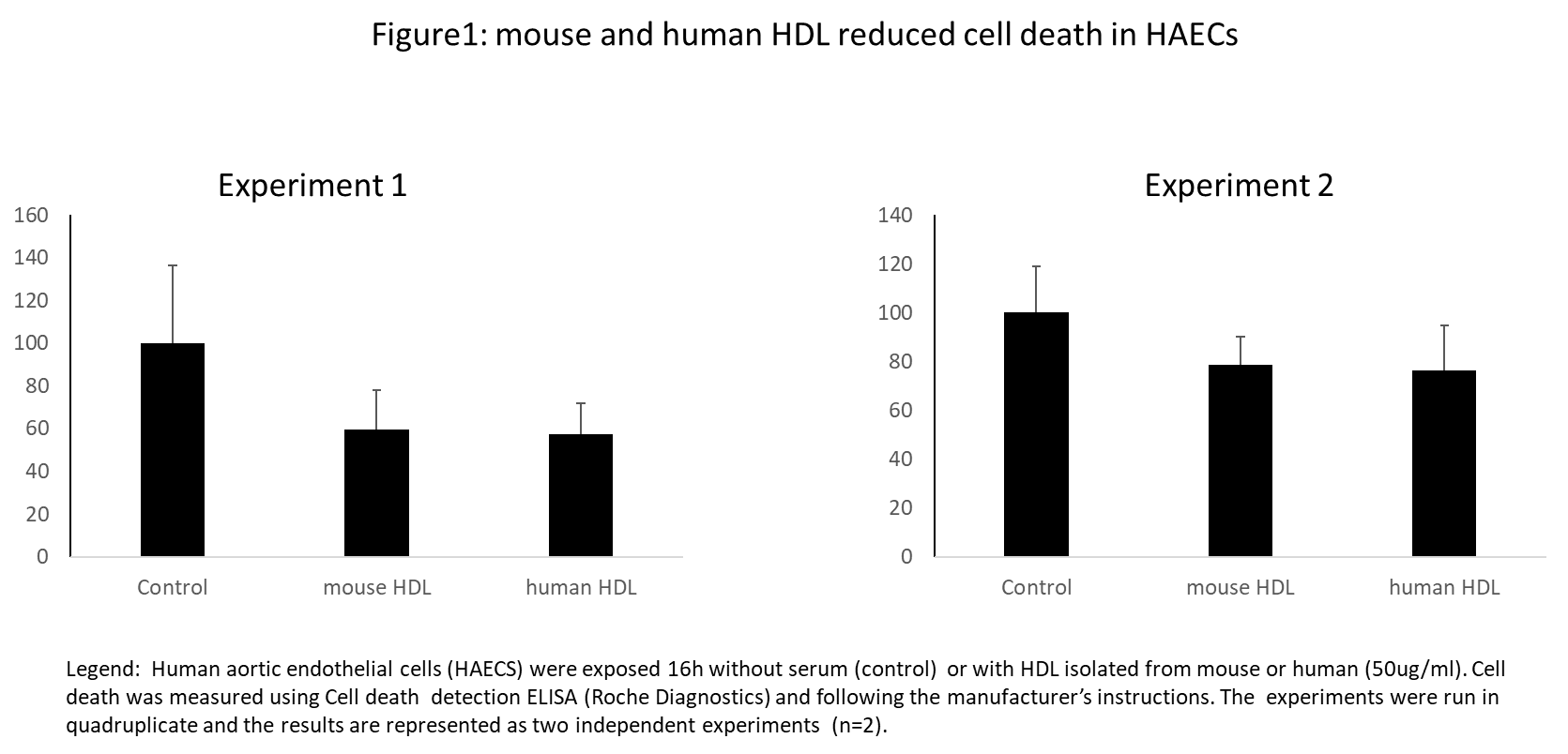
**

**S2 Fig: Both mouse and human HDL reduced cell death in HAECs.**

Human aortic endothelial cells (HAECs) were exposed 16h without serum (control) or with HDL isolated from mouse or human (50μg/ml). Cell death was measured using cell death detection ELISA (Roche Diagnostics) and following the manufacturer’s instructions. The experiments were run in quadruplicate and the results are represented as 2 independent experiments (n=2).
