# Supplementary material for: HDL protects against myocardial ischemia reperfusion injury via miR-34b and miR-337 expression which requires STAT3
Source: PLoS One. 2019 Jun 20;14(6):e0218432. doi: 10.1371/journal.pone.0218432 (PMC6586303; doi:10.1371/journal.pone.0218432)
Supplement: S3 Fig — (DOCX) [file pone.0218432.s004.docx]

**Supporting Information**

S3 Fig


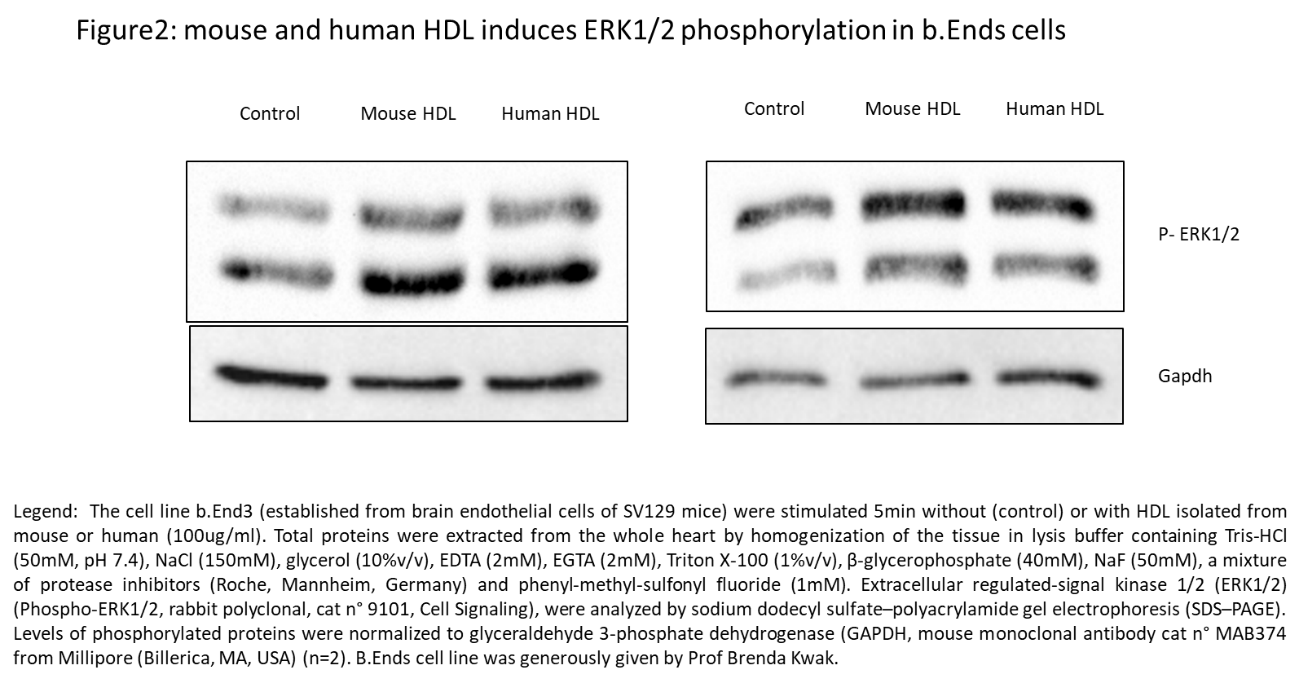


**S3 Fig: Both mouse and human HDL induced ERK1/2 phosphorylation in bEnd.3 cells.**

The cell line bEnd.3 (established from brain endothelial cells of SV129 mice) were stimulated 5min without (control) or with HDL isolated from mouse or human (100μg/ml). Total proteins were extracted from the whole heart by homogenization of the tissue in lysis buffer (Tris-Hcl (50mM, pH 7.4), NaCl (150mM), glycerol (10%v/v), EDTA (2mM), EGTA (2mM), Triton X-100 (1%v/v), ß-glycerophosphate (40mM), NaF (50mM), mix of protease inhibitors (Roche) and phenyl-methyl-sulfonyl fluoride (1mM)). Extracellular regulated-signal kinase 1/2 (ERK1/2) (phospho-ERK1/2 rabbit polyclonal antiboby, cat #9101, Cell Signaling) phosphorylation was analyzed by sodium dodecyl sulfate-polyacrylamide gel electrophoresis (SDS-PAGE). Levels of phosphorylated proteins were normalized to glyceraldehyde 3-phosphate dehydrogenase (GAPDH mouse monoclonal antibody, cat #MAB374, Millipore). bEnd.3 cell line was generously given by Prof Brenda Kwak.
